# Resisting the Effects of Neoliberalism on Public Policy: Comment on "Implementing Universal and Targeted Policies for Health Equity: Lessons From Australia"

**DOI:** 10.34172/ijhpm.2022.7354

**Published:** 2022-06-22

**Authors:** Dennis Raphael, Toba Bryant

**Affiliations:** ^1^School of Health Policy and Management, York University, Toronto, ON, Canada.; ^2^Faculty of Health Sciences, Ontario Tech University, Oshawa, ON, Canada.

**Keywords:** Neoliberalism, Liberal Welfare States, Health Equity, Social Determinants, Australia

## Abstract

Fisher and colleagues carefully review the extent to which health equity goals of availability, affordability, and acceptability have been achieved in the areas of national broadband network policy and land-use policy, in addition to the more traditional areas of primary healthcare and Indigenous health in Australia. They consider the effectiveness of policies identified as either universal, proportionate-universal, targeted or residualist in these areas. In this commentary we suggest future areas of inquiry that can help inform the findings of their excellent study. These include the impacts of Australia being a liberal welfare state and how acceptance of neoliberal approaches to governance makes the achieving of health equity in these four policy areas difficult.

## Introduction

 Fisher and colleagues are to be commended for providing us with a state-of-the-art analysis of the ins and outs of implementing universal and targeted policies for health equity in Australia.^1^ One innovative aspect of this study was their examination of the implementation of national broadband network policy and land-use policy, in addition to the more traditional areas of primary healthcare and Indigenous health. They carefully review the extent to which these policies have been able to achieve health equity goals of availability, affordability, and acceptability through implementation of policies identified as either universal, proportionate- universal, targeted or residualist.

 Overall, they conclude that performance on the three dimensions of health equity in health of availability, affordability, and acceptability was only partially successful in the primary healthcare, broadband access, and land-use policy areas. They were somewhat more successful in the Indigenous health domain. Their *Closing the Gap* case study, for example, showed that equity of service access for Aboriginal and Torres Strait Islander communities warrants both forms of targeting to ensure cultural safety of universal services and to strengthen stand-alone community-led services, programs and strategies. They conclude that residualist policies do not promote equity of access to resources and see value in universal and proportionate-universal approaches that are tailored to specific health policy contexts.

 The article is very rich in concepts and findings and provides a good introduction to many key issues in the health policy and equity realm. These concepts include the idea of health equity, different forms of public policy action to achieve health equity, and concrete examples of how these processes play out in these four areas of health-related public policy activity in Australia. The methodology also provides a state-of-the-art model for both new and experienced researchers employing qualitative case studies involving the mapping of policy structures, engaging with grey literatures to track policy debate and change, and carrying out in-depth interviews with key informants from government and non-government agencies and independent experts.

 Fisher et al also identify a dimension with real implications for understanding how public policy can promote health equity: centralized versus devolved governance structures. They suggest that devolved structures would be more effective for implementing equity but do not provide much detail as to why governmental authorities are reluctant to implement such processes. They also suggest that funding tends to be targeted project funding rather than agency funding, a feature also related to governance structures. Also noteworthy is that their focus on primary care and Indigenous health services is primarily reactive, saying little about the economic and political forces that drive the healthcare related needs of vulnerable social groups. Readers are urged to read their article which offers many insights into problematic approaches to promoting health equity through health policy. Our comments are provided primarily to suggest future areas of inquiry that can help inform the findings of their excellent study.

## Welfare States, Neoliberalism, and Redistribution

 The authors mention how recent ideological trends have shaped public policy development and implementation: “Since the 1980s, the rise of neoliberal politics favouring reduced state intervention in capitalist markets has seen some retreat from universalism and revival of selective, targeted approaches” (p. 2). Key aspects of adopting neoliberal approaches to governance involve limiting social spending and coverage by the public healthcare system.^2^ Australia ranks 26th of 28 among Organization for Economic Cooperation and Development (OECD) nations in social spending and 24th of 36 OECD nations in managing income inequality.^3,4^ Only 68% of healthcare spending is public spending with 20% of spending being out of pocket, amongst the highest figures among OECD nations.^5^

 But, the authors do not detail nor explain how this neoliberal trend has shaped the funding and accountability mechanisms governments have implemented for health and social services organization and delivery. And it is important to note that while these developments have been apparent across all forms of the welfare state they have been especially noticeable in liberal welfare states of which Australia is a good example.^6^

 There have also been macro-level effects that influence the health and well-being of equity-seeking groups. Garret^7^ identifies six dimensions of macro-level neoliberal governance which clearly have relevance for the issues discussed by Fisher and colleagues: (1) overturning embedded liberalism which regulated entrepreneurial and corporate activities at the end of World War II until the mid-1970s; (2) the re-configuration of the state to better serve the interests of capital; (3) patterns of income and wealth distribution which benefit the rich at the expense of most others; (4) increasing insecurity and precariousness; (5) a rise in mass incarceration resulting from increases in crime related to growing income inequality and precariousness; and (6) a strategic pragmatism by which governing authorities are willing to stray from the tenets of neoliberalism when faced with natural or economic crises. Potential responses to these are presented in the following sections.

## Neoliberalism and Transformations in the Provision of Health and Social Services

 There are aspects of neoliberal ideology which directly affect the organization and delivery of health and social services similar to issues raised by Fisher and colleagues. Baines^8^ argues that neoliberalism’s valorizing of “the private market, economic rationalism, and individual, rather than collective, responsibility for social and individual ills” (p. 12) has affected the non-profit sector in which healthcare and social services are delivered. Such processes would explain much of the centralized top-down, targeted funding favored by Australian authorities which Fisher and colleagues decry as reducing the capacity of service providers to meet the needs of particular populations by limiting the flexibility required to address their diverse needs, thereby limiting the quality of service provision:

 “*In the nonprofit workplace, the neoliberal drift saturates managerial models such as new public management and other forms of performance and outcome management. These approaches purportedly coach employees in “best practices” and increase professional competencies, but in the name of increasing efficiencies and removing waste and error, these processes standardize work practices, reduce or remove employee discretion, and increase the pace and volume of work as well as the risk of staff burnout, demoralization, and workplace illness and injury*”^7^ (p. 12).

 Baines^8,9^ documents the effects of these transformations of social services in Canada. There has been a shift from secure to project funding which require service agencies to justify funding through the use of concrete and narrow metrics drawn from business models such as New Public Management. New Public Management has led to service standardization, excessive concern with metrics, and a decline in advocacy and community mobilization efforts. All of these trends were mentioned in Fisher and colleagues’ article and make achieving the goal of devolving governance processes for promoting equity more difficult.

## The Way Forward

 Fisher and colleagues suggest that reporting research evidence can convince authorities to devolve decision-making to local authorities and agencies, thereby promoting health equity:

 “*Similarly, our CTG case study indicates that devolved governance at a regional or local scale can play a role in effective implementation of targeted policies, again by flexibly tailoring actions to meet local communities’ needs and goals. A systemic shift to use of such structures could overcome some of the aforementioned weaknesses of targeted funding practices such as short-termism, duplication and excessive regulatory demands”* (p. 9).

 But if these funding practices are driven by the forces we have mentioned above, additional actions are required. Interestingly, the most developed literature on forms of resistance to these trends comes from the social services rather than the healthcare literature.^10^ In regard to social services practice, Weinberg and Banks^11^ identify three forms of resistance available to the social work profession which may be relevant to those working in a variety of public policy areas: political, social, and ethical. Political resistance involves opposing problematic public policies that inequitably distribute resources and create vulnerability. It also includes resisting broader phenomena such as economic globalization, unfettered capitalism, or even capitalism itself. Social resistance can involve opposing discriminatory norms and practices by joining social movements such as Black Lives Matter, the labour movement, or other human rights organizations. Ethical resistance is focused on individual actions, and in the context of social work practice, would be about resisting institutional practices that undermine the organization and delivery of social services.

 A commitment to equity is an ethical stance. For the most part we would expect healthcare workers, advocates for broadband internet access, and equitable land use planning would subscribe to ethical principles of social justice, equity, and human rights. If economic and political forces such as acceptance of neoliberal approaches to governance around the making of public policy are driving the findings reported in this study, then these forces must be resisted. While argued in relation to social service organization and delivery in the Nordic nations, Kamali and Jonsson’s^12^ compelling statement of how these forces can be resisted may very well be relevant to promoting health equity across a range of public policy domains in Australia and elsewhere:

 “*In this, critical social work should encourage cooperation with a number of agents: people in need of social work interventions; political parties defending the revitalisation of the welfare state; trade unions of social workers committed to the global ethics and values of social work; civil society solidary organisations; and NGOs engaged in improving the living conditions of people and in counteracting increasing inequalities, marginalisation, racism and exclusion. This is the only way in which critical social work can remain committed to its core values, to social justice, to solidarity and to its emancipatory mission and potential”* (p. 267).

## Ethical issues

 Not applicable.

## Competing interests

 Authors declare that they have no competing interests.

## Authors’ contributions

 Both authors contributed equally to this work.
